# Cytokine expression and mortality risk among COVID-19 hospitalized patients over 60 years of age in a referral hospital in Cartagena, Colombia

**DOI:** 10.1016/j.heliyon.2024.e29028

**Published:** 2024-04-02

**Authors:** Remberto Ramos-González, Eder Cano-Pérez, Steev Loyola, Rita Sierra-Merlano, Doris Gómez-Camargo

**Affiliations:** a Departamento de Medicina Interna, Facultad de Medicina, Universidad de Cartagena, Cartagena, Colombia; b Grupo de Investigación UNIMOL, Facultad de Medicina, Universidad de Cartagena, Cartagena, Colombia; c Doctorado en Medicina Tropical, Facultad de Medicina, Universidad de Cartagena, Cartagena, Colombia; d Facultad de Medicina, Universidad Peruana Cayetano Heredia, Lima, Peru

**Keywords:** Cytokines, COVID-19, Death, Prognosis, Intensive care unit

## Abstract

**Background:**

Cytokine dysregulation in COVID-19 patients aged over 60 has been associated to adverse outcomes. While serum levels have been studied, cellular expression, particularly in Afro-Colombians, remains understudied. This research aims to describe cytokine expression in peripheral blood leukocytes and its association with adverse outcomes in COVID-19 patients aged over 60 at Cartagena's referral hospital.

**Methods:**

A cohort study was conducted, encompassing severe and critical cases of COVID-19 between November 2021 and February 2022. At baseline, the cellular expression level of cytokines IL-2, IL-4, IL-6, IL-10, TNF-α and IFN-γ was assessed using flow cytometry. Additionally, various biochemical, hematological, and coagulation markers were evaluated. The main outcome was time to death.

**Results:**

Among the 50 enrolled participants, the median age was 76.5 years, 60% were male, 60% were admitted to the ICU, and 42% died. Lactate dehydrogenase and hemoglobin were the only markers that differed between fatal and surviving cases. Regarding cytokines, the level of IL-6 expression was associated with an increased risk of death. Specifically, a one percent increase in the expression was associated with a 7.3% increase in the risk of death. Stratifying the analysis by death and ICU admission, the median expression level remained high in fatal cases who were admitted to the ICU.

**Conclusions:**

Our findings revealed a significant association between high cellular expression levels of IL-6 and an increased risk of mortality. These results provide valuable scientific insights that could inform the prioritization of case management, providing especially advantageous for the vulnerable Afro-Colombian group.

## 1 Introduction

The COVID-19 pandemic has had a devastating impact on the healthcare systems of low-to middle-income countries. In Colombia, as of June 2023, there have been over 6 million confirmed cases and more than 142,000 deaths from COVID-19. Among the affected age groups, individuals over 60 years old have experienced significant consequences [1]. Although transmission rates remain relatively low, the sustained global and local transmission continues to result in additional deaths that may be linked to the emergency and predominance of viral variants with enhanced immune system evasion capabilities and the likelihood of incomplete vaccine protection [2]. By identifying factors associated with a higher risk of death, it becomes possible to develop and implement intervention strategies aimed to mitigate the impact on vulnerable groups in the early stage of the disease.

Numerous global studies have examined demographic and clinical factors associated with severity and death from COVID-19. Factors consistently associated with negative clinical outcomes include older age, hypertension, diabetes, obesity, and cardiovascular, respiratory, and renal diseases [[3], [4], [5], [6], [7], [8]]. In addition, being male has been identified as a risk factor for both death and complications [7]. While limited research has been conducted in the Colombian population, local reports indicate that older age [9,10], male sex [10], and comorbidities such as ischemic heart disease and chronic obstructive pulmonary disease (COPD) are associated with an increased risk of admission to the intensive care unit (ICU) as well as an increased risk of death [9].

Among the laboratory markers, elevated levels of ferritin, C-reactive protein, D-dimer, troponins, and lactate dehydrogenase, alongside reduced levels of monocytes, lymphocytes, and platelets, have been identified as factors associated with a higher risk of complications and death from COVID-19 [3,11]. Similarly, increased levels of interleukin-1 (IL-1), IL-6, IL-7, IL-10, tumor necrosis factor-alpha (TNF-α), and reduced expression of interferons (IFNs) have been associated with negative outcomes in individuals with COVID-19 [12]. These altered markers contribute to an exacerbated inflammatory response that promotes multiorgan deterioration and the onset of acute respiratory distress syndrome (ARDS), which eventually leads to death [12].

Previous studies have explored the correlation between serum levels of pro-inflammatory (IL-2, IFN-γ, TNF-α) or anti-inflammatory cytokines (IL-4 and IL-10) with clinical or negative outcomes in individuals with COVID-19 [[13], [14], [15]]. However, to our knowledge, there has been a scarce evaluation of the cellular expression of cytokines in vulnerable and high-risk groups, particularly in individuals over 60 years old. Thus, the research aim was to characterize the expression levels of IL-2, IL-4, IL-6, IL-10, TNF-α, and IFN-γ in peripheral blood leukocytes and to assess the relationship between expression levels and the risk of death among individuals older than 60 years old who had COVID-19 and were admitted to the unique referral hospital in Cartagena de Indias, Colombia.

## 2 Material and methods

### 2.1 Study design and population

A prospective cohort study was conducted at Hospital Universitario de Cartagena between November 2021 and February 2022. The hospital is located in the city of Cartagena (Coordinates: 10.40042, −75.50352), which is the capital of the Bolivar department in Colombia. The hospital is a public institution that provides medium- and high-complexity services to Cartagena's population and patients referred from healthcare centers in the department of Bolivar. Its wide range of services includes emergency care, outpatient care, surgery, hospitalization, and ICU, among others. Because of its complexity, the hospital was designated as a referral center in the Bolivar department [16]. During the study period, the Omicron variant of SARS-CoV-2 was predominant in Cartagena [1].

During the study period, individuals aged 60 years or older, diagnosed with severe or critical COVID-19 at hospital admission [17], and confirmed by RT-PCR or antigen testing, were eligible for enrollment as meeting the inclusion criteria established in this study. Individuals who participated in experimental studies evaluating pharmacological interventions (such as vaccines or drugs) against COVID-19 were excluded. Taking into consideration these predefined criteria, a total of 66 cases were identified during the study period, of which 16 (22.9%) declined to participate in the study. Therefore, the findings described in this manuscript correspond to 50 cases. The research project was approved by the ethics committee of the Universidad de Cartagena (No. 2022-4). Written informed consent was obtained from all cases to access their medical records and to collect two peripheral blood samples.

### 2.2 Variables and data collection

*Outcome:* The outcome of interest was time to death. Time zero was defined as the date of hospital admission, and the end of follow-up was determined by either death or hospital discharge. Information was collected from the medical records.

*Covariates:* Baseline demographic data, including sex and age, as well as clinical variables such as comorbidities (hypertension, cardiovascular disease [coronary artery disease and/or heart failure], diabetes, asthma, COPD, cerebral vascular accident [CVA], chronic kidney disease, cancer, rheumatoid arthritis, and Alzheimer's disease), were collected. Additionally, at baseline, serum levels of lactate dehydrogenase (IU/L), ferritin (mg/L), C-reactive protein (mg/dL), sodium (mEq/L), and potassium (mEq/L) were quantified. Total leukocytes (103/μL), relative percentage (%), and absolute count (103/μL) of neutrophils and lymphocytes, neutrophil/lymphocyte ratio (NLR), hemoglobin (g/dL), mean corpuscular volume (MCV; ft), mean corpuscular hemoglobin (MCH; pg), mean corpuscular hemoglobin concentration (MCHC; g/dL), and platelets (103/μL) in whole blood were also measured. In plasma, the activated partial thromboplastin time (APTT) and prothrombin time (PT)/international normalized ratio (INR) were estimated. All aforementioned biomarkers were obtained from the medical records, and their abnormalities were determined considering the reference values used in the hospital. For cytokine quantification, peripheral blood samples were collected within the first 24–48 h after hospital admission in tubes containing EDTA. The quantification of IL-2 (Ref: 12-7029-42), IL-4 (Ref: 48-7049-42), IL-6 (Ref: 46-7069-42), IL-10 (Ref: 53-7108-42), TNF-α (Ref: 56-7349-42), and IFN-γ (Ref: 47-7319-42) in peripheral blood leukocytes was conducted using intracellular reagents and antibodies (Invitrogen eBioscience) following manufacturer's recommendations on an Attune NxT flow cytometer (ThermoFisher; catalog number: A24861). The expression of each cytokine was reported as the percentage of positive events among the total evaluated events (10,000 events by case). The elapsed time from symptom onset to hospital admission was estimated based on the reported symptom onset date, while ICU admission information was collected from the medical records.

### 2.3 Statistical analysis

Categorical variables were summarized using frequencies, while continuous variables were presented using medians (p50) and interquartile ranges (IQR). Bivariate relationships were evaluated by Fisher's exact test, Mann-Whitney *U* test, and Kruskal-Wallis test with ties. The Spearman's rank correlation test with Bonferroni adjustment was used to construct a correlation matrix with data from all analyzed biomarkers (biochemical, hematological, coagulation, and cytokines). The Kaplan-Meier estimator and log-rank test were used to construct survival curves and evaluate differences between them, respectively. Generalized linear models with a Poisson distribution family and a logarithmic function were constructed to calculate crude and adjusted incidence rate ratios (IRR) and their 95% confidence intervals (95% CI). The adjusted models (aIRR) included sex and age as confounders. Four post hoc groups were defined based on ICU admission and death: i) admitted to ICU and died; ii) not admitted to ICU and died; iii) admitted to ICU and survived; and iv) not admitted to ICU and survived. Statistical significance was defined as p < 0.05, and all analyses were performed using Stata v.17.0 (StataCorp. 2021. Stata Statistical Software: Release 17. College Station, TX: StataCorp LLC) and RStudio.

## 3 Results

**Fig. 1:**
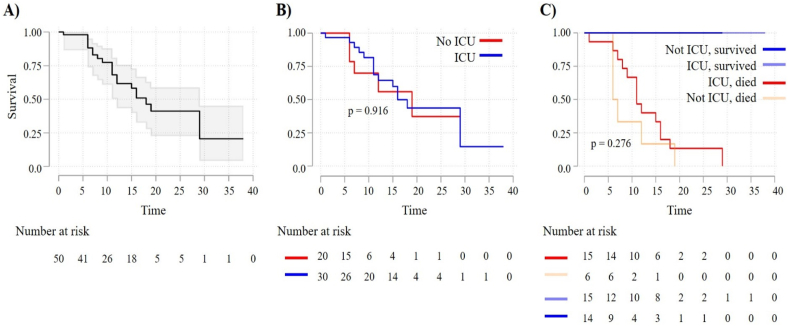
Kaplan-Meier survival curves for hospitalized COVID-19 cases. Survival curves were plotted for the entire cohort (Panel A), by the ICU admission (panel B), and by the ICU admission and death (Panel C). The log-rank was used to test differences in survival curves. Risk tables (number of cases at risk) are presented below plots.

Among the individuals enrolled in the study, 60.0% (30/50) were male, the median age was 76.5 years (IQR: 12.0), and the median elapsed time from the onset of the illness to hospital admission was 5 days (IQR = 5). Sixty percent of the enrolled were admitted to ICU, and the cumulative incidence of death was 42.0% (21/50). Fig. 1A depicts the survival function for the entire cohort. Sex (p = 0.154), age (p = 0.371), and ICU admission (p = 0.243) were not associated with death.

Hypertension (74.0%), diabetes (24.0%), and Alzheimer's disease (12.0%) were the most frequent comorbidities, while asthma (6.0%), cancer (6.0%), cardiovascular disease (6.0%), COPD (6.0%), stroke (4.0%), chronic kidney disease (4.0%), and arthritis (4.0%) were the least frequent. None of the comorbidities was significantly associated with death.

**Fig. 2:**
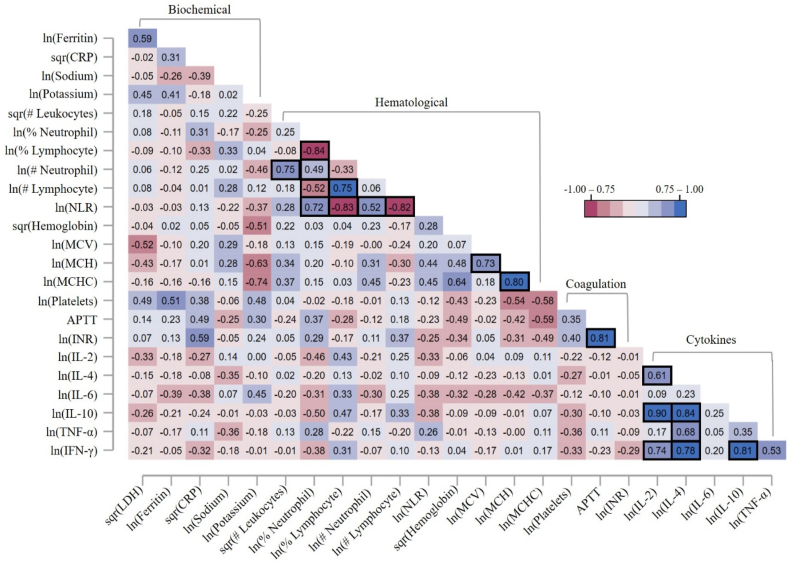
Correlation matrix between cytokines and biochemical, hematological, and coagulation biomarkers. Values were transformed using ln or sqr functions. Correlations were estimated using Spearman's rank test and coefficients were Bonferroni corrected. Significant (p < 0.005) correlation coefficients are highlighted using black-bordered squares.

The biomarkers examined here are summarized in Table 1. Biochemical, hematological, and coagulation biomarkers did not significantly differ between survivors and deceased individuals, except for lactate dehydrogenase and hemoglobin (Table 1). Specifically, deceased individuals exhibited higher levels of lactate dehydrogenase (p = 0.008) and lower levels of hemoglobin (p = 0.036) compared to survivors. Regarding cytokines, only IL-6 levels were significantly higher in deceased individuals compared to survivors (p = 0.008). The correlation matrix depicting the relationships among all assessed biomarkers is displayed in Fig. 2. As expected, multiple hematological biomarkers were correlated (Fig. 2). Additionally, significant correlations were observed between various cytokines (Fig. 2).

**Table 1:** Biological markers and cytokine levels. Concentrations or levels are presented using median (IQR).

Biomarkers	Normal value range	All cases	Death		p-value
			No	Yes	
Biochemical					
LDH (UI/L)	105–333	334.0 (143.0)	311.0 (127.0)	404.5 (99.5)	0.007
Sodium (mEq/L)	135–145	137.5 (7.0)	137.0 (7.5)	138.0 (6.0)	0.388
Potassium (mEq/L)	3.6–5.2	4.2 (1.0)	4.0 (1.0)	4.3 (0.9)	0.148
C-reactive protein (mg/dL)	<0.3	12.5 (13.9)	13.2 (13.9)	6.7 (15.4)	0.476
Ferritin (mg/L)	M: 18.0–270.0 W: 18.0–160.0	798.5 (1084.0)	734.5 (775.5)	1235.5 (1005.0)	0.069
Hematological					
Leucocytes (10^3^/μL)	5.0–10.0	9.2 (6.5)	9.0 (6.5)	9.7 (7.1)	0.329
Neutrophils (%)	40–60	83.8 (13.2)	83.1 (12.8)	89.5 (15.8)	0.152
Neutrophils (10^3^/μL)	1.4–7.0	7.5 (5.8)	7.5 (4.9)	7.3 (6.9)	0.464
Lymphocytes (%)	20–40	7.8 (9.3)	7.8 (8.2)	8.4 (14.2)	0.927
Lymphocytes (10^3^/μL)	0.7–3.1	0.8 (0.6)	0.7 (0.5)	0.8 (1.4)	0.349
NLR	1.5–1.6	10.9 (12.6)	10.9 (10.4)	10.5 (17.4)	0.776
Hemoglobin (g/dL)	M: 13.2–16.6 W: 11.6–15.0	12.2 (2.2)	12.5 (2.1)	11.7 (2.2)	0.036
MCV (ft)	80.0–100.0	92.3 (6.7)	92.4 (6.1)	91.7 (5.9)	0.182
MCH (pgr)	27.0–33.0	30.0 (2.7)	30.1 (1.9)	29.9 (3.1)	0.752
MCHC (g/dL)	32.0–36.0	32.5 (1.3)	32.5 (1.2)	32.5 (2.0)	0.847
Coagulation					
Platelet (10^3^/μL)	150.0–400.0	192.0 (122.0)	191.0 (117.0)	204.5 (176.5)	0.295
APTT (sec)	30.0–40.0	31.0 (5.5)	29.5 (5.9)	31.8 (4.5)	0.142
INR	<1.1	1.0 (0.1)	1.0 (0.1)	1.0 (0.2)	0.709
Cytokines					
IL-2	N.E.	28.4 (9.0)	26.7 (6.9)	29.2 (9.1)	0.651
IL-4	N.E.	29.6 (10.3)	30.2 (10.0)	29.5 (9.0)	0.859
IL-6	N.E.	23.1 (10.1)	20.2 (8.4)	28.3 (7.0)	0.008
IL-10	N.E.	27.2 (7.2)	24.6 (6.6)	28.4 (6.5)	0.302
TNF-α	N.E.	16.9 (2.3)	16.9 (1.7)	16.9 (2.5)	0.891
IFN-γ	N.E.	24.8 (8.6)	23.7 (8.2)	27.1 (11.2)	0.542

Note: The reference values are those established by the hospital. Missing data for multiple variables: 13 for APTT and INR; 12 for Ferritin; 11 for Lactate dehydrogenase; 10 for C-reactive protein; 7 for Potassium; 6 for Sodium; 3 for MCV; and 1 for all hematological markers. N.E.: Not established.

The risk of death increased by 0.4% for every 1 IU/L increase in lactate dehydrogenase. This finding was marginally significant in both crude (IRR: 1.004; p = 0.083) and adjusted analysis (aIRR: 1.004; p = 0.103). Conversely, the risk of death decreased by 10.3–12.4 % for every 1 g/dL increase in hemoglobin. This finding was marginally significant in the crude analysis (IRR: 0.876; p = 0.116) and not significant in the adjusted analysis (aIRR: 0.897; p = 0.234). In contrast, an increase of one percentage unit in IL-6 expression was associated with an increased risk of death by 7.3% (IRR: 1.073; p = 0.047). The risk, although marginally significant, remained higher when controlling for sex and age (aIRR: 1.064; p = 0.107). Despite the observed correlation between multiple cytokines (Fig. 2), cytokine levels other than IL-6 did not exhibit significant associations with the risk of death.

**Fig. 3:**
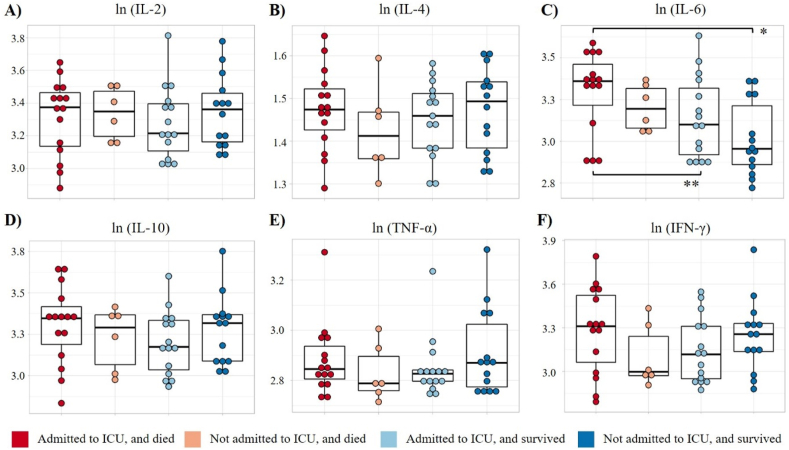
Expression levels of cytokines stratified by ICU admission and death. A) IL-2. B) IL-4. C) IL-6. D) IL-10. E) TNF-α. F) IFN-γ. Box plots show values transformed using the ln function. *p < 0.010, **p < 0.05.

In the post hoc analysis, no significant differences were observed in the survival curves among the analyzed groups (Fig. 1B and C). The expression levels of IL-2, IL-4, IL-10, TNF-α, and IFN-γ were comparable across all evaluated groups (Fig. 3). Interestingly, distinct and significant variations were noted when assessing IL-6 levels among the analyzed groups. Specifically, individuals who were admitted to the ICU and subsequently died exhibited significantly higher levels of IL-6 compared to those who survived (Fig. 3C).

## 4 Discussion

Here, we present the assessment of intracellular expression levels of multiple cytokines in leukocytes collected from individuals older than 60 years with severe or critical COVID-19. Our findings suggest a correlation between elevated IL-6 levels during the first 48 h of hospitalization and an increased risk of death. Notably, we observed significantly higher levels in those patients who were admitted to the ICU and subsequently died as compared to patients who, regardless of their admission to the ICU, survived. While our evaluation focused on intracellular expression rather than serum IL-6 levels, our findings are consistent with multiple previously published studies describing cohorts of similar size to the one described here. For instance, Guirao et al. in Spain reported an increase in serum IL-6 levels among ICU-admitted cases and cases that subsequently died [17]. In Germany, Herold et al. reported the relationship between high IL-6 levels and the need for mechanical ventilation among COVID-19 cases [18]. Similarly, In China, elevated serum IL-6 levels have been documented in patients who died of COVID-19 compared to surviving cases [14,19]. Furthermore, Liu et al. reported an increase of IL-6, IL-10, IL-2, and IFN-γ in severe COVID-19 cases [15]. Moreover, it has been described that the sustained elevation of IL-6 and other cytokines is associated with death in severe COVID-19 cases [14]. In Colombia, Delgado-Murcia et al., in a series of 24 cases, described significantly higher IL-6 concentrations in hospitalized cases compared to those that did not require hospitalization [13].

The cellular expression level of IL-6 in individuals older than 60 years with COVID-19 could have a potential predictive value for death, according to our findings. Specifically, we observed a 7.3% increase in risk of death for every one percentage point increase in IL-6 levels. The limited sample size might account for the lack of significance in the adjusted analysis; however, it is important to highlight that both the direction and magnitude of the risk remained consistent when controlling for sex and age. A previous study has explored the utility of quantifying serum IL-6 for predicting mortality [18]. However, the prognostic or predictive value of cellular expression of IL-6 is still unknown. In light of the findings described here, further studies are required to systematically evaluate and validate the potential of IL-6 cellular expression levels in predicting death or other outcomes.

Numerous cytokines have been implicated in the severity of COVID-19 [[20], [21], [22], [23], [24], [25]]. As with IL-6, IL-10 has been proposed as a predictive marker, suggesting that measuring IL-6 and IL-10 levels could be useful in assessing the prognosis of patients with COVID-19 [[20], [21], [22]]. Increased levels of other cytokines such as IFN-γ and TNF-α have also been observed in severe cases compared with mild cases [[23], [24], [25]]. In contrast, cytokines such as IL-2 and IL-4 have been described as not associated with severity [26]. In our study, we did not identify significant differences in the expression of IFN-γ, TNF-α, IL-10, IL-2, and IL-4 among the evaluated groups. The lack of differences could be explained by the enrollment of patients with severe or critical disease, rather than cases without severity, as well as unstudied factors that may have been related to the disease caused by the circulating variant during the study period.

Lactate dehydrogenase is an enzyme involved in energy production and is found in almost every cell, thus, it is considered a marker of tissue damage and inflammation [27]. Previous studies have reported elevated lactate dehydrogenase levels in deceased COVID-19 cases [28]. Moreover, it has been described that elevated lactate dehydrogenase levels at hospital admission are associated with an increased risk of in-hospital mortality [29]. Conversely, it has been previously suggested that patients with anemia frequently develop severe COVID-19 [30]. In our study, we observed significantly elevated LDH levels in deceased patients, surpassing the reference values. Additionally, fatal cases exhibited notably lower hemoglobin levels. Overall, our findings are consistent with those previously reported [[27], [28], [29], [30]]. Regarding other laboratory parameters, consistent associations have been documented between increased levels of C-reactive protein [31,32], ferritin [32,33], INR [33], and NLR [34] in patients with negative COVID-19 outcomes, such as severity or death. However, these associations were not observed in our study. The absence of these relationships may be attributed to the limited cohort size, resulting in insufficient statistical power.

This study is subject to several limitations. First, the generalizability of our findings may not be appropriated for unstudied population groups. Previous studies have suggested that genetic factors and other characteristics play a significant role in various COVID-19 outcomes [35]. Therefore, the specific characteristics of the enrolled Afro-Colombian population may limit the utility of our findings to other population groups. Second, the lack of healthy individuals in our study did not allow us to determine “baseline” levels of cell expression. Third, we did not assess the kinetics of cytokine levels over the follow-up period. Fourth, our outcomes may have been influenced by factors such as the availability of beds, patients' refusal to be admitted to the ICU, or other logistical and structural factors that affect access to specialized clinical management. Fifth, the intracellular levels of cytokines might not precisely mirror the post-secretory levels found in serum. Since serum cytokine levels were not assessed here, we suggest that future investigations include measurements for both cellular and serum cytokines. This approach would offer a more comprehensive understanding of cytokine production in individuals with severe or critical COVID-19. Despite these limitations, this study possesses several noteworthy strengths. To the best of our knowledge, this study represents one of the initial attempts to explore multiple biological markers in the Afro-descendant population of the Colombian Caribbean, particularly in subgroups with high mortality rates and a poor prognosis. Based on the study design, it is plausible to propose that intracellular expression of IL-6 could serve as an early marker with the potential to predict mortality. Further studies are necessary to validate the role of cellular expression in clinical outcomes and the need for specialized ICU care.

Here, we provide a comprehensive analysis of the biochemical, hematological, coagulation, and cellular expression profiles of various cytokines in a cohort of severe/critical COVID-19 patients aged over 60 years who were hospitalized at the unique referral hospital in Cartagena. Among the multiple markers analyzed, we observed a significant association between high cellular expression levels of IL-6 and an increased risk of death from COVID-19. The implications of these findings are noteworthy, particularly in the context of resource-limited settings, as they provide valuable insights to guide prioritization in case management decisions.

## Ethics statement

This study was evaluated and approved by the Ethics Committee of the University of Cartagena. Written informed assent or consent was requested from all participants.

## Funding

This research and the APC were funded by Universidad de Cartagena under the program “Plan de Fortalecimiento de Doctorado de la Universidad de Cartagena”; UDC-2022.

## Data availability statement

Data will be made available on request.

## CRediT authorship contribution statement

**Remberto Ramos-González:** Writing – original draft, Methodology, Investigation, Formal analysis, Data curation, Conceptualization. **Eder Cano-Pérez:** Writing – original draft, Methodology, Investigation, Formal analysis. **Steev Loyola:** Writing – original draft, Methodology, Formal analysis, Data curation. **Rita Sierra-Merlano:** Writing – review & editing, Supervision, Resources, Project administration, Methodology, Funding acquisition, Conceptualization. **Doris Gómez-Camargo:** Writing – review & editing, Supervision, Resources, Project administration, Funding acquisition, Conceptualization.

## Declaration of competing interest

The authors declare that they have no known competing financial interests or personal relationships that could have appeared to influence the work reported in this paper.
